# Dietary Phytochemicals Targeting NRF2 Against Skin Cellular Senescence: Mechanistic Insights and Potential for Functional Food Development

**DOI:** 10.3390/biology15010039

**Published:** 2025-12-25

**Authors:** Yi Liu, Ruiqi Wang, Hanqing Liu, Zhigang Tu

**Affiliations:** 1 School of Life Sciences, Jiangsu University, Zhenjiang 212013, China; liuyi1112003@163.com (Y.L.); rachelwang23@163.com (R.W.); 2 School of Pharmacy, Jiangsu University, Zhenjiang 212013, China

**Keywords:** dietary phytochemicals, cellular senescence, NRF2, skin aging, functional foods, antioxidants, phenolics

## Abstract

Skin aging is driven largely by oxidative stress, chronic inflammation, and mitochondrial dysfunction, processes closely linked to cellular senescence and declining NRF2 activity. Numerous dietary phytochemicals—such as curcumin (from turmeric), resveratrol (from grapes), sulforaphane (from cruciferous vegetables), zerumbone, and salvianolic acid B—abundant in fruits, vegetables, herbs, and traditional food sources, exhibit potent antioxidant and anti-inflammatory properties. This review systematically elucidates the molecular mechanisms by which these compounds mitigate skin aging, primarily through modulating the NRF2 signaling pathway. We further integrate insights from clinical trials of NRF2-targeting agents to inform the translational potential of these dietary bioactives. Molecular docking analyses confirm that these food-derived compounds interact directly with the KEAP1-NRF2 complex, promoting NRF2 activation. Transcriptomic analyses of skin-related datasets (GSE35160, GSE71910, GSE185129) further validate the downregulation of key NRF2-regulated cytoprotective genes (e.g., FTH1, FTL, HMOX1, SLC7A11) involved in antioxidant defense and the suppression of pro-inflammatory mediators. Based on this mechanistic foundation, we discuss the translational potential of these food-derived bioactives and the rationale for their future incorporation into skin-health-promoting nutraceuticals. We highlight how these food-derived phenolics and other bioactives may be incorporated into functional foods or nutraceuticals to support skin health from within, offering a dietary strategy to delay aging. We acknowledge that key translational challenges, such as oral bioavailability and optimal formulation, require further investigation. Further research is warranted to bridge these mechanistic insights into effective human applications.

## Simple Summary

Skin aging is a natural process that becomes more visible due to factors like sun exposure, pollution, and unhealthy lifestyles, leading to wrinkles, loss of elasticity, and dull skin. This review explores how certain natural compounds found in everyday foods—such as turmeric, grapes, broccoli, ginger, and traditional herbs—can help slow down skin aging by targeting cellular pathways. These dietary ingredients work by activating a protective protein in our cells called NRF2, which helps reduce damage caused by stress and inflammation, and supports the health of skin cells. We summarize scientific evidence showing that these food-derived compounds can enhance the skin’s natural defenses, improve its appearance, and delay signs of aging. This research supports the idea that what we eat can directly influence how our skin ages, offering a natural and accessible approach to maintaining skin health through diet and functional foods. Further studies may help develop effective food-based products for long-term skin care.

## 1. Introduction

The skin, as the largest organ of the human body, accounts for approximately 16% of body weight and is composed of three layers: the epidermis, dermis, and subcutaneous adipose tissue [1,2]. Notably, the skin is also a key endocrine organ, responsible for the synthesis of vitamin D (cholecalciferol) upon ultraviolet (UV) exposure. Vitamin D plays a critical role in maintaining skin homeostasis by supporting epidermal barrier integrity, modulating immune function, and regulating cell proliferation and differentiation, all of which are relevant to the skin aging process [3]. Skin aging is a progressive process of functional decline, primarily characterized by reduced regenerative capacity, diminished elasticity, increased wrinkling, and decreased collagen synthesis [4,5]. Skin aging is driven by a combination of intrinsic and extrinsic factors, which collectively disrupt skin homeostasis, leading to impaired barrier function, delayed wound healing, chronic inflammation, and increased susceptibility to cancers [6,7]. It is important to note that human skin is a highly heterogeneous organ. Its properties and aging process vary significantly based on genetic factors, such as Fitzpatrick skin type (which determines pigmentary phenotype and UV response), leading to distinct clinical and molecular aging phenotypes. Intrinsic aging, governed by chronological biological processes, manifests as fine wrinkles and epidermal thinning [8,9]. In contrast, extrinsic aging, primarily caused by ultraviolet radiation, smoking, and environmental pollutants, leads to more pronounced morphological changes, including deep wrinkles, loss of elasticity, and pigmentary alterations [10,11].

Cellular senescence represents a stable cell cycle arrest, serving as a fundamental hallmark of aging [12]. It can be triggered by diverse stimuli, including DNA damage, telomere dysfunction, oncogene activation, and organelle stress, and plays pivotal roles in tumor suppression, tissue repair, and developmental processes [13]. Cellular senescence is a finely regulated biological process characterized by distinct phenotypic alterations, such as changes in cell morphology, metabolism, epigenetic regulation, and gene expression [14,15]. Accumulating evidence underscores cellular senescence as a key driver of skin aging. Senescent cells progressively accumulate in chronologically aged and photoaged skin, contributing to age-related cutaneous alterations and pathological manifestations [16,17].

Studies have demonstrated that protein levels of nuclear factor erythroid 2-related factor 2 (NRF2) exhibit a significant age-dependent decline [18]. This reduction leads to decreased expression of downstream antioxidant genes regulated by NRF2 [19,20], rendering cells more susceptible to oxidative damage and thereby accelerating the aging process. Traditional dietary systems and herbal medicines provide a vast repository of natural ingredients for health maintenance [13,21]. Its rich bioactive compounds provides invaluable natural resources for drug development [22]. Recent studies have identified that multiple bioactive components derived from common food sources and dietary plants can modulate the NRF2 pathway to influence cellular senescence and exhibit remarkable efficacy in ameliorating skin aging, positioning them as promising candidates for nutraceutical development [23,24].

## 2. Molecular Mechanisms of Skin Aging Induced by Cellular Senescence

### 2.1. The Critical Roles of Cellular Senescence in Skin Aging

Unlike other organs, the skin, as the body’s outermost organ, is persistently exposed to diverse external stimuli [25]. It is subjected not only to intrinsic aging processes but also to damage from environmental factors, particularly ultraviolet (UV) radiation, chemical agents, and thermal stressors [26,27]. The elasticity and firmness of skin primarily depend on the synthesis of collagen and elastin. When dermal fibroblasts undergo senescence, their proliferative capacity declines alongside reduced production of collagen and elastin, leading to skin laxity and wrinkle formation [28]. Similarly, epidermal cellular senescence impairs proliferative ability, compromising epidermal repair and regeneration. These changes manifest as epidermal thinning, reduced cell density, slowed turnover, weakened skin barrier, increased susceptibility to environmental damage, and delayed wound healing [29,30]. Chronic low-grade inflammation exacerbates skin aging through the senescence-associated secretory phenotype (SASP), particularly via pro-inflammatory cytokines like IL-6 and TNF-α secreted by senescent cells [27,31]. These cytokines not only accelerate skin aging but may also contribute to dermatological conditions including xerosis, erythema, and cutaneous hypersensitivity [31].

### 2.2. Triggering Mechanisms of Cellular Senescence in Skin Tissue

#### 2.2.1. Oxidative Stress

Cellular senescence in skin tissue is closely associated with oxidative stress. Reactive oxygen species (ROS), including superoxide anion (O_2_•^−^), hydrogen peroxide (H_2_O_2_), and hydroxyl radical (OH•), are highly reactive byproducts of cellular metabolism that play crucial roles in signal transduction, proliferation, and immune responses [32,33]. However, excessive ROS production induces oxidative stress, damaging cellular components such as DNA, proteins, and lipids, thereby accelerating cellular senescence [33,34]. As the outermost protective barrier of the human body, skin tissue tends to actively produce higher levels of excessive ROS.

UV radiation is an important exogenous factor contributing to ROS production in the skin. Both ultraviolet A (UVA) and ultraviolet B (UVB) radiation can induce ROS generation, leading to structural and functional damage in skin cells. UVB is primarily absorbed by the epidermis, triggering ROS production and DNA damage [35]. Additionally, various chemicals—derived from air pollutants and cosmetic ingredients—can stimulate ROS production in skin cells [36].

ROS can directly attack DNA, causing DNA damage, which is a key inducer of cellular senescence [37]. DNA damage activates the DNA damage response (DDR), subsequently triggering signaling pathways such as p53/p21 and p16/Rb, ultimately leading to cell cycle arrest and cellular senescence [38]. Furthermore, telomeres—protective structures at chromosome ends—are also susceptible to ROS-induced damage and shortening, thereby promoting cellular senescence [37,39].

#### 2.2.2. Inflammation

ROS can activate the mitogen-activated protein kinase (MAPK) signaling pathway, including extracellular signal-regulated kinase (ERK), c-Jun N-terminal kinase (JNK), p38, etc. [40,41]. Activated MAPK further stimulates the NF-κB signaling pathway [42]. Upon activation, NF-κB upregulates the expression of pro-inflammatory cytokines, such as tumor necrosis factor-alpha (TNF-α) and interleukin-1 beta (IL-1β), thereby exacerbating inflammatory responses [43]. Inflammatory factors, particularly certain SASPs, can induce or reinforce cellular senescence [44,45]. These factors not only affect senescent cells themselves but also exert paracrine effects on neighboring cells, leading to the propagation of inflammation and acceleration of senescence [44,45].

#### 2.2.3. Mitochondrial Dysfunction

Mitochondria serve as the primary intracellular source of ROS, while excessive ROS may damage mitochondrial DNA (mtDNA). Such mtDNA damage leads to mitochondrial dysfunction, which in turn promotes excessive ROS production, ultimately establishing a vicious cycle of “ROS–mtDNA-damage–mitochondrial dysfunction” [46,47].

Mitochondrial dysfunction plays a pivotal role in cellular senescence through complex mechanisms. First, oxidative stress represents a major pathway through which mitochondrial dysfunction contributes to cellular senescence [48]. Second, mitochondrial dysfunction affects cellular metabolism, resulting in insufficient energy supply that further promotes cellular senescence [49]. Additionally, cellular senescence is associated with the accumulation of mtDNA mutations and the decline of respiratory chain function [50].

**Figure 1:**
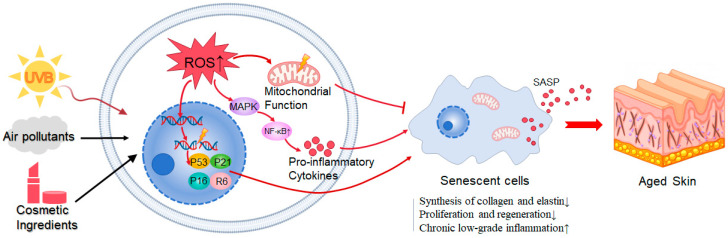
Cellular senescence induces skin aging through multiple molecular pathways.

As depicted in Figure 1, cellular senescence drives skin aging through oxidative stress, inflammation (elevated pro-inflammatory cytokines like IL-6 and TNF-α), and mitochondrial dysfunction. These processes collectively reduce collagen and elastin synthesis, impair skin cell proliferation and regeneration, and amplify chronic low-grade inflammation via SASP, ultimately leading to skin thinning, wrinkle formation, and barrier dysfunction.

## 3. Regulatory Role of the NRF2 Pathway in Skin Cellular Senescence

The NRF2 pathway plays a crucial regulatory role in skin cellular senescence by exerting anti-senescence effects through multiple coordinated mechanisms: primarily by upregulating antioxidant gene expression to enhance ROS scavenging capacity and thereby alleviate cellular oxidative stress; secondly, by inhibiting the activation of the NF-κB signaling pathway to reduce the secretion of pro-inflammatory cytokines (including IL-6 and TNFα); and finally by improving mitochondrial function through activation of mitochondrial respiratory activity and consequent reduction in ROS generation.

### 3.1. Scavenging Reactive Oxygen Species (ROS)

NRF2 serves as a pivotal transcriptional regulator of cellular redox homeostasis, orchestrating the activation of cytoprotective genes to counteract basal and stress-induced oxidative damage [51,52]. The activity of NRF2 is tightly regulated through its interaction with KEAP1 (Kelch-like ECH-associated protein 1) [53,54]. Under oxidative stress, NRF2 dissociates from KEAP1, translocates to the nuclei, and induces the expression of antioxidant response element (ARE)-dependent genes, including superoxide dismutase 1 (SOD1), heme oxygenase-1 (HO-1), NAD(P)H quinone dehydrogenase 1 (NQO1), and catalase (CAT), etc.

SOD1, a critical antioxidant enzyme, scavenges ROS by catalyzing the disproportionation of superoxide radicals into hydrogen peroxide and molecular oxygen [55,56]. HO-1, a stress-inducible enzyme, plays a central role in maintaining cellular redox homeostasis through multiple antioxidant mechanisms [57]. It catalyzes the degradation of heme into biliverdin, ferrous iron, and carbon monoxide (CO) [58]. Biliverdin is subsequently reduced to bilirubin, a potent antioxidant capable of neutralizing ROS, by biliverdin reductase [59].

**Figure 2:**
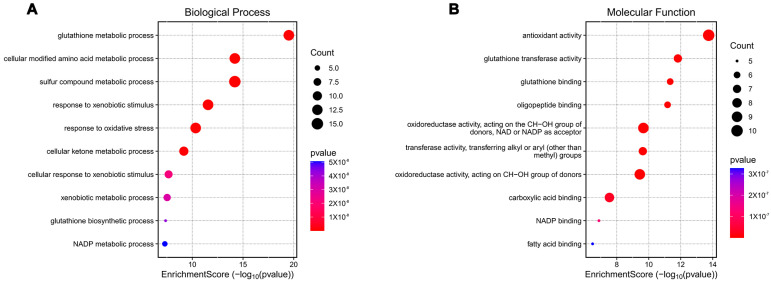
Gene Ontology (GO) enrichment analysis of differentially expressed genes (DEGs) in GSE35160 [60]. (**A**) Biological processes (GO-BP) enriched in DEGs. (**B**) Molecular functions (GO-MF) enriched in DEGs.

Schäfer et al. [60] generated transgenic mouse models expressing a constitutively active Nrf2 mutant (caNrf2) in keratinocytes to explore the role of Nrf2 in skin biology. Transcriptomic data (GSE35160) were analyzed using GEO2R to compare gene expression profiles between the caNrf2 and wild-type groups. Differentially expressed genes (DEGs) were selected with the thresholds of |logFC| ≥ 1 and a *t*-test *p*-value < 0.05. Functional enrichment analysis of DEGs were performed using the GO-KEGG pathway module on the online platform https://www.bioinformatics.com.cn (last accessed on 10 December 2024), followed by visualization (Figure 2). Notably, biological processes associated with ROS clearance, including the glutahione metabolic process and response to oxidative stress, were ranked first and fifth, respectively (Figure 2A). Among the top five molecular function categories, four were directly linked to oxidative stress elimination (Figure 2B).

**Figure 3:**
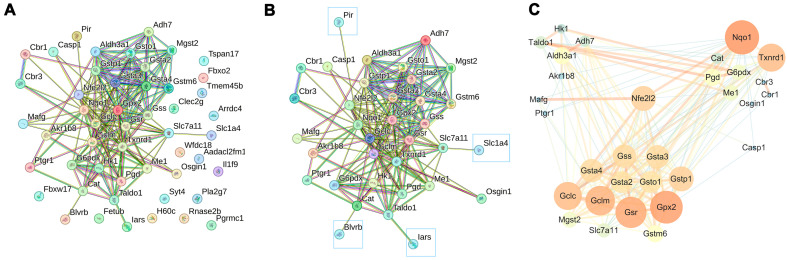
Protein–protein interaction (PPI) network analysis of DEGs in GSE71910. (**A**) STRING network of all DEGs. (**B**) STRING network of key DEGs. (**C**) Core PPI network of high-confidence interactions.

Using the same transgenic mouse models, Rolfs et al. performed transcriptome resequencing of mouse skin (GSE71910). Differential gene expression analysis was conducted using GEO2R to compare the caNrf2-expressing group with wild-type controls. Applying thresholds of |logFC| ≥ 0.5 and a *t*-test *p*-value < 0.05, we identified 49 DEGs. These DEGs, along with NRF2 (encoded by Nfe2l2), were analyzed using STRING 2.0 (total *n* = 50 genes) with default parameters. As demonstrated in Figure 3, the protein–protein interaction network revealed a complex interaction network comprising 35 genes, while the remaining 15 genes appeared as isolated nodes.

In Figure 3, proteins with only one interaction (including Pir, Blvrb, Iars, and Slc1a4; indicated by framed boxes) were removed using Cytoscape 3.10.2. Network analysis revealed that 30 proteins formed distinct functional clusters centered on NRF2, with the largest cluster (13 proteins) primarily involved in glutathione synthesis and metabolism. Glutathione plays a critical role in redox homeostasis by directly scavenging ROS and serving as an essential substrate for glutathione peroxidase (GPx)-mediated reduction of hydrogen peroxide and organic peroxides. Additional functional clusters included a NADPH-generating cluster comprising Pgd, G6pdx, and Me1; carbonyl compound detoxification enzymes Cbr1 and Cbr3 that metabolize endogenous and exogenous carbonyl compounds; and redox-regulatory proteins (Nqo1, Txnrd1, Cat, and Osgin1) that collectively protect cells from oxidative damage through their complementary antioxidant functions. The results demonstrate NRF2′s central role in coordinating multiple antioxidant defense systems through these protein interaction networks.

**Figure 4:**
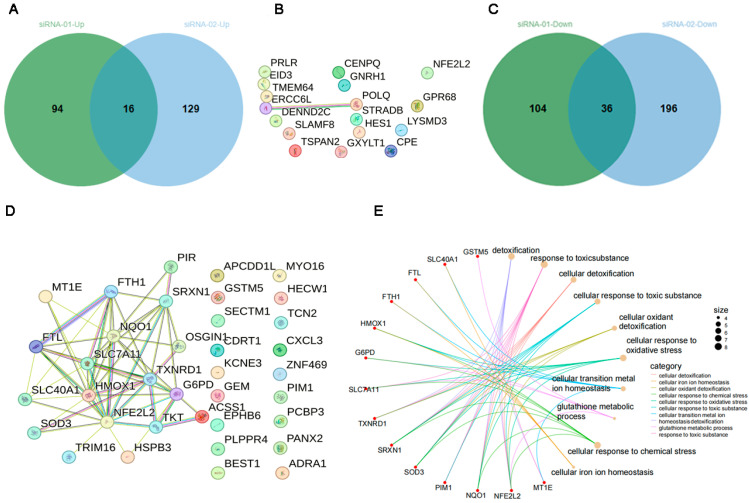
Transcriptomic analysis of DEGs in GSE185129. (**A**,**B**) Up-regulated DEGs. (**C**,**D**) Down-regulated DEGs. (**E**) Biological process (BP) network (cnetplot) of GO analysis for all DEGs.

Salamito and Ruggiero established primary skin fibroblast cultures from three young donors and performed transfections with either scrambled siRNA or NRF2-targeting siRNAs (GSE185129). Transcriptomic profiling was conducted using the Illumina NextSeq 500 platform (Homo sapiens). We performed differential gene expression analysis using GEO2R, comparing NRF2 siRNA-treated samples with scrambled siRNA controls (selection criteria: |logFC| ≥ 1, *t*-test *p*-value < 0.05). Following DEG identification in each siRNA group, common differentially expressed genes were identified through comparative analysis of both up-regulated and down-regulated gene pools (Figure 4A–D).

As demonstrated in Figure 4A, sixteen proteins showed consistent up-regulation across both siRNA groups. However, STRING database analysis of these proteins together with NFE2L2 revealed no significant protein–protein interactions (Figure 4B). Literature review indicated that most of these genes are not established NFE2L2 transcriptional targets. These findings suggest that the observed transcriptional up-regulation of these genes likely represents experimental false positives rather than biologically relevant NRF2-mediated regulation.

In contrast, STRING database analysis of the 36 down-regulated proteins (including NFE2L2) revealed that 18 proteins formed a functionally interconnected network (Figure 4C,D). Comprehensive literature review confirmed that 13 of these proteins (excluding the four boxed proteins) are well-characterized NFE2L2 transcriptional targets involved in either antioxidant defense or xenobiotic detoxification pathways. Functional annotation (Table S1) demonstrated their specific roles: (1) FTH1, FTL, heme oxygenase 1 (HMOX1), and SLC7A11 coordinately regulate cellular iron metabolism; (2) SRXN1 and TXNRD1 maintain thioredoxin redox system integrity; and (3) the remaining proteins mediate diverse antioxidant mechanisms, including ROS scavenging and electrophile detoxification.

GO enrichment analysis of the 52 commonly regulated genes (both up- and down-regulated) revealed significant enrichment in related biological processes, including cellular detoxification, cellular response to oxidative stress, and cellular transition metal ion homeostasis (Figure 4E). Figure S1 demonstrates consistent enrichment patterns between the STRING and GO-BP algorithms.

Collective analysis of the above–mentioned functional datasets (GSE35160, GSE71910, and GSE185129) reveals that NRF2 pathway activation provides comprehensive protection against oxidative stress by reducing ROS accumulation, strengthening cellular antioxidant defense systems, and ultimately preventing oxidative stress-induced senescence in skin cells [61,62].

### 3.2. Inflammation Suppression

In addition to mitigating oxidative stress and thereby attenuating inflammatory responses [63], the NRF2 pathway suppresses inflammation through multiple mechanisms targeting the activation of NF-κB, a key transcriptional regulator of inflammation.

NF-κB orchestrates the expression of various pro-inflammatory cytokines. NRF2 inhibits NF-κB signaling through several distinct pathways: (1) Under basal conditions, NF-κB remains inactive through binding with IκB. Upon stimulation, IκB undergoes phosphorylation and degradation, releasing NF-κB for nuclear translocation and transcriptional activation. NRF2 blocks this process by inhibiting IκB phosphorylation and degradation [64,65,66]; (2) NRF2 may competitively bind co-activators (e.g., CBP) to suppress NF-κB activity [67]; (3) NRF2-induced HO-1 expression interferes with NF-κB activation, reducing production of pro-inflammatory cytokines (e.g., IL-1β, IL-6, and TNFα) [68,69].

Furthermore, NRF2-regulated enzymes demonstrate anti-inflammatory properties: (1) NQO1 suppresses IL-1β, IL-6 and TNFα production, mitigating inflammatory damage [69]; (2) SOD1 inhibits NF-κB activation and subsequent pro-inflammatory cytokine production [70], thereby efficiently decreasing inflammatory responses; (3) SOD1 modulates caspase-1 activity, thereby regulating IL-1β generation.

**Figure 5:**
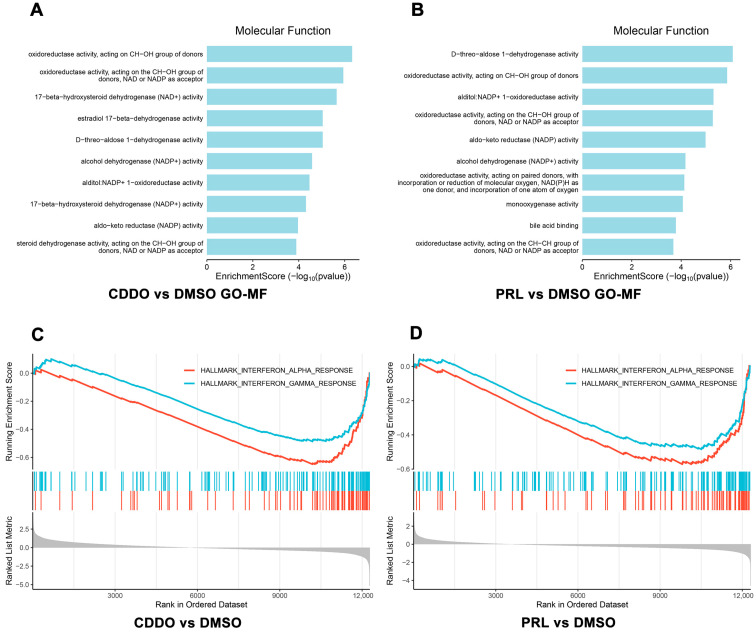
Functional and enrichment analyses of GSE241267 transcriptomic data. (**A**,**B**) GO-MF enrichment of DEGs. (**C**,**D**) Gene set enrichment analysis (GSEA) of all protein-coding transcripts.

Barakat et al. treated human keratinocytes with either CDDO-Me (an electrophilic NRF2 activator) or PRL-295 (a non-electrophilic NRF2 activator), using DMSO as the vehicle control. The original RNA-seq data were obtained from the GEO database (GSE241267). Following data preprocessing, only protein-coding genes were retained for downstream analysis. GO-KEGG pathway enrichment analysis of DEGs between CDDO-Me and DMSO-treated groups (|logFC| ≥ 1, *t*-test *p* < 0.05) revealed significant enrichment of pathways associated with redox regulation and NAD^+^/NADP^+^ reduction, consistent with NRF2′s known biological functions (Figure 5A). A comparable enrichment pattern was observed in the PRL-295 vs. DMSO comparison (Figure 5B). Furthermore, GSEA of all filtered protein-coding genes demonstrated that both NRF2 activators significantly suppressed inflammatory pathways, highlighting their anti-inflammatory potential in keratinocytes (Figure 5C,D).

### 3.3. Improvement of Mitochondrial Functions

Firstly, mitochondrial dysfunction is closely associated with ROS accumulation. NRF2 activation induces various antioxidant enzymes to scavenge ROS, thereby mitigating oxidative damage to mitochondria and maintaining their normal functions.

Secondly, NRF2 activation promotes mitochondrial biogenesis [71,72]. The specific mechanisms include: (1) NRF2 interacts with PGC-1α (peroxisome proliferator-activated receptor gamma coactivator 1-alpha) to coordinately regulate mitochondrial biogenesis [73]. As a key regulator of mitochondrial biogenesis, PGC-1α activates multiple transcriptional factors to promote mitochondrial DNA replication and protein synthesis. NRF2 enhances PGC-1α expression and potentiates its role in mitochondrial biogenesis. (2) Emerging evidence indicates NRF2 can directly upregulate mitochondrial transcription factor TFAM to facilitate mitochondrial DNA replication and transcription [74].

Thirdly, mitophagy—a selective autophagy process for removing damaged mitochondria—is essential for maintaining mitochondrial quality and function [75]. The NRF2 pathway regulates mitophagy through multiple mechanisms: NRF2 activation enhances autophagy, a crucial cellular clearance mechanism for damaged organelles and proteins to maintain homeostasis and delay aging [76,77]. By promoting autophagic activity, NRF2 facilitates the removal of cytotoxic debris, thereby attenuating senescence-associated damage and inflammation [78,79].

## 4. Regulation of NRF2 Activity in Skin Tissue

### 4.1. Canonical Regulatory Pathways of NRF2

Under normal physiological conditions, KEAP1 forms a complex with NRF2 and sequesters it in the cytoplasm [80]. KEAP1, serving as an adaptor protein for E3 ubiquitin ligase, facilitates NRF2 ubiquitination and subsequent proteasomal degradation [80,81,82]. When cells are exposed to oxidative stress or electrophilic compounds, specific cysteine residues on KEAP1 may get modified [33,81,83]. Such modifications may induce conformational changes in KEAP1, leading to NRF2 release from the KEAP1 complex. The liberated NRF2 then translocates into the nucleus [84,85].

Within the nucleus, NRF2 forms heterodimers with small musculoaponeurotic fibrosarcoma (sMAF) and binds to AREs [33]. This binding activates transcription of downstream target genes including HO-1 and NQO1, which encode proteins involved in regulating redox metabolism, inflammation, and protein homeostasis.

### 4.2. Non-Canonical Regulatory Pathways of NRF2

In addition to KEAP1, NRF2 is also regulated by other upstream signaling molecules. SIRT1 stabilizes NRF2 and promotes its nuclear translocation by mediating NRF2 deacetylation, thereby upregulating downstream antioxidant targets and enhancing antioxidant capacity. Conversely, SIRT1 deficiency impairs NRF2 transcriptional activity and compromises antioxidant function [86]. Phosphorylation kinases, such as MAPK and ERK, can directly or indirectly phosphorylate NRF2, increasing its activity, stability, and transcriptional potency [87,88].

### 4.3. Phytomedicines Targeting the NRF2 Pathway in Skin Aging

Several dietary polyphenolic compounds, including resveratrol (found in grapes and berries), curcumin (the primary curcuminoid in turmeric), and genistein (a major isoflavone in soybeans), have been reported to activate the NRF2 pathway through direct or indirect mechanisms. These food-derived compounds effectively delay senescence in various skin cell types and ameliorate skin aging in murine models [26,89,90].

Traditional diets and food-derived botanicals have long been recognized for their distinctive health-promoting and anti-aging properties [91]. Accumulating evidence indicates that extracts and compounds from these dietary sources can effectively delay skin aging and enhance cutaneous function through multiple mechanisms, including antioxidant activity and stimulation of cellular regeneration [92].

Sulforaphane (SFN), an isothiocyanate derived from cruciferous vegetables such as broccoli and cabbage, ameliorates skin aging and cellular senescence through NRF2 pathway activation [93]. In dermal fibroblasts (DFs) and HaCaT keratinocytes, aloe-derived nanoparticles facilitate NRF2 nuclear translocation, activate intracellular ROS scavenging pathways, mitigate UV-induced oxidative stress and DNA damage, suppress cellular senescence, and inhibit β-galactosidase activity and SASP elevation. In vivo, aloe-derived exosome-like nanoparticles reduce malondialdehyde (MDA) while enhancing SOD levels in murine skin, thereby delaying photoaging [94].

*Nigella sativa* L. seed (commonly known as black seed) extract demonstrates anti-melanocyte senescence effects by downregulating KEAP1 levels and upregulating NRF2 expression, subsequently activating antioxidant genes (HO-1 and NQO1) [95]. Zerumbone, a sesquiterpene from the ginger family plants, exhibits anti-photoaging properties in UVA-irradiated human dermal fibroblasts by elevating total and nuclear NRF2 levels, activating the NRF2/ARE defense pathway, and scavenging excess ROS [96]. Salvianolic acid B (Sal-B), a key water-soluble compound derived from the herb *Salvia miltiorrhiza* (Danshen), upregulates NRF2 expression and downstream antioxidant genes while inhibiting the NF-κB inflammatory pathway, thereby counteracting UVB-induced skin aging through ROS clearance and anti-inflammatory mechanisms [97,98].

Galangin, a flavonoid found in galangal and propolis, reverses H_2_O_2_-induced fibroblast senescence via the SIRT1-PGC-1α-NRF2 axis and alleviates UVB-induced photodamage in C57BL/6J nude mice [26]. Puerariae lobatae radix (PLR), the root of kudzu vine, protects against UVB-induced skin aging by functioning as a REVERBα antagonist—it enhances BMAL1 expression, subsequently elevating NRF2 transcriptional activity and protein levels, upregulating antioxidant gene expression, and ultimately mitigating UVB-induced skin aging through ROS scavenging and senescence suppression.

**Figure 6:**
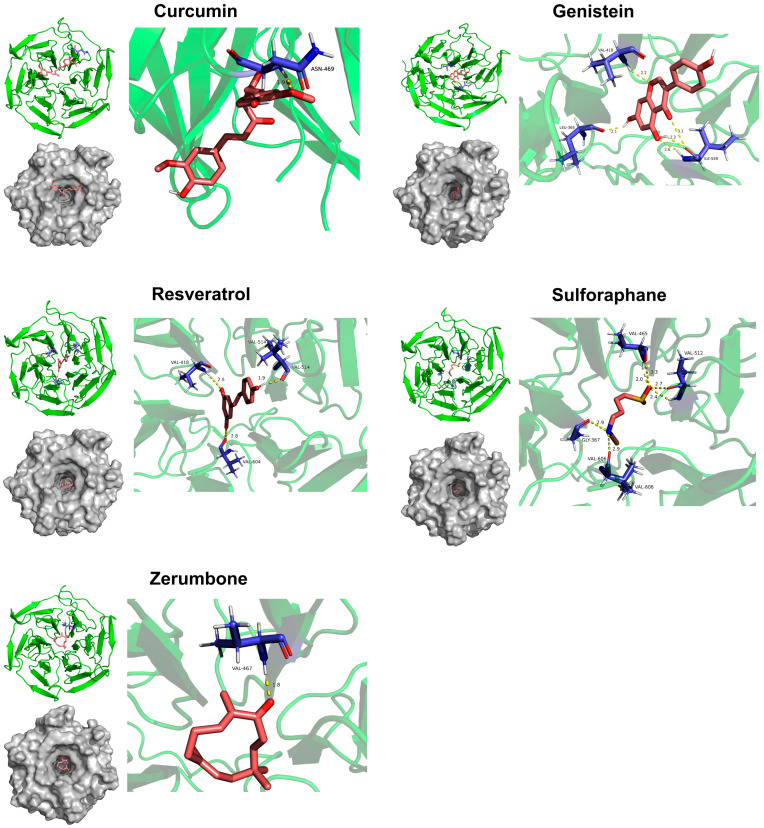
Molecular docking analysis of dietary phytochemical compounds (curcumin, genistein, resveratrol, sulforaphane, and zerumbone) with the KEAP1 Kelch domain. The binding conformations and interactions of each compound within the KEAP1 binding pocket are shown.

Molecular docking analysis of the dietary compounds curcumin, genistein, resveratrol, sulforaphane, and zerumbone with the KEAP1-NRF2 binding complex revealed that all compounds occupied the concave binding pocket of the KEAP1 Kelch domain, which normally interacts with the N-terminal region of NRF2 (Figure 6). Based on calculated binding energies, the compounds exhibited differential binding affinities: curcumin and sulforaphane showed weaker interactions with KEAP1, while genistein, resveratrol, and zerumbone demonstrated stronger binding (Table S2). These findings indicate that these dietary phytochemicals may exert dual protective mechanisms—serving not only as oxidizable substrates to mitigate oxidative stress but also as direct activators of NRF2 through competitive binding to KEAP1.

## 5. Discussion

While the mechanistic evidence supporting NRF2-activating phytochemicals in skin aging is compelling, translating these insights into effective dietary interventions benefits from alignment with existing clinical knowledge on NRF2 modulation. A comprehensive review of trials involving established NRF2-inducing agents (e.g., sulforaphane, oltipraz) confirms the pharmacological relevance of this pathway in humans and offers key translational insights [99]. Notably, the induction of NRF2 target genes (e.g., NQO1, HMOX1) in human trials, though consistent, often exhibits a limited dynamic range, suggesting a saturable dose–response that underscores the importance of precise dosing. Furthermore, significant inter-individual variability in biomarker response—influenced by genetics, microbiome composition, and baseline physiology—highlights the context-dependent nature of efficacy. These clinical observations, together with the absence of a universal standalone biomarker for NRF2 activity, collectively define a practical framework for future research. Therefore, the established clinical profile of NRF2 activation provides a strategic foundation for developing dietary interventions for skin health, wherein these clinical insights inform the rational design of functional foods with targeted efficacy.

**Figure 7:**
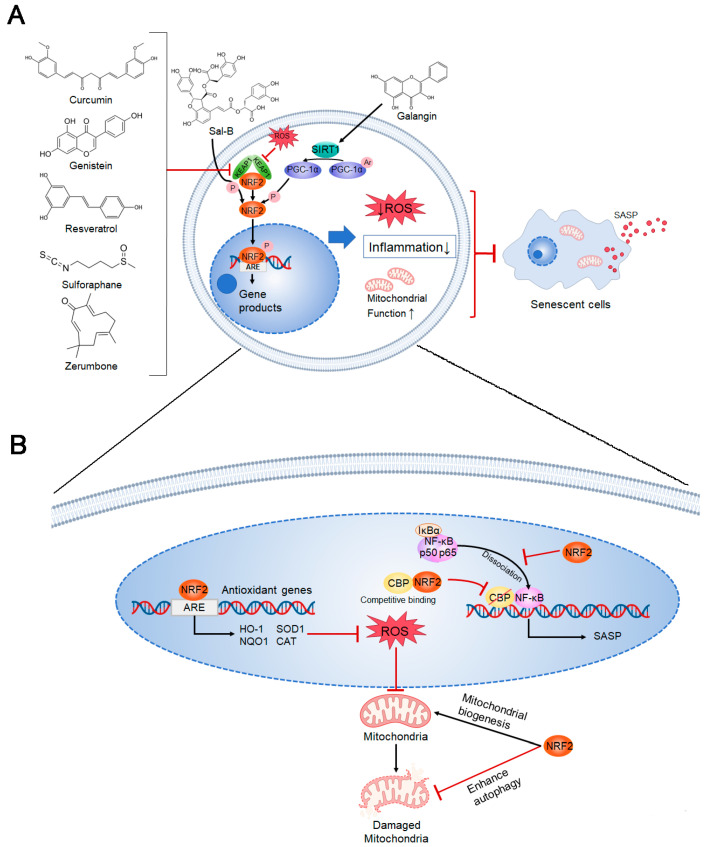
NRF2-dependent regulation of skin cellular senescence by herbal compounds. (**A**) Regulatory mechanisms of dietary compounds on NRF2 activation. (**B**) Specific mechanisms of NRF2 in regulating cellular senescence in skin cells.

This review highlights the complex mechanisms by which NRF2 influences skin aging (Figure 7). NRF2 activation reduces oxidative stress by upregulating antioxidant enzymes (e.g., HO-1, SOD1, NQO1, CAT) to scavenge ROS, which may inadvertently disrupt cellular redox homeostasis and contribute to senescence under chronic conditions. Multiple dietary phytochemicals target this pathway: curcumin, genistein, resveratrol, sulforaphane, and zerumbone competitively bind the KEAP1-NRF2 complex, while Sal-B directly upregulates NRF2 expression. NRF2 suppresses inflammation by inhibiting NF-κB signaling and pro-inflammatory cytokine production, yet prolonged suppression may impair immune surveillance. Galangin uniquely modulates this balance via SIRT1-PGC-1α-NRF2 axis activation.

Conversely, NRF2 enhances mitochondrial function by promoting mitochondrial biogenesis (via PGC-1α) and mitophagy, thereby mitigating senescence-associated mitochondrial dysfunction. The age-related decline in NRF2 activity exacerbates oxidative damage, inflammation, and mitochondrial failure, collectively accelerating skin aging. Notably, these compounds derived from food and botanicals exhibit tissue-specific effects: sulforaphane and zerumbone show particular efficacy in UV-protection by enhancing NRF2/ARE pathway activation in dermal fibroblasts, whereas curcumin and resveratrol demonstrate broader anti-senescence effects across epidermal and dermal cells. It is noteworthy that the biological outcomes of NRF2 activation are highly context-dependent. For instance, sustained, constitutive activation of NRF2 in fibroblasts can drive cellular senescence and promote a cancer-associated fibroblast phenotype, highlighting the importance of achieving moderate (e.g., via dietary intake) rather than prolonged activation [100].

However, translating these promising mechanistic findings into practical applications faces a series of translational challenges. The mechanistic and pre-clinical evidence discussed herein primarily derives from models that may not fully capture the diversity of human skin. A critical, yet understudied, translational gap is the potential influence of skin type (e.g., Fitzpatrick phototype I–VI) on the efficacy of NRF2-targeting phytochemicals. For instance, skin with higher constitutive melanin may have different baseline oxidative stress and antioxidant reservoir levels, which could modulate the response to exogenous NRF2 activators. Conversely, individuals with lower melanin content (more susceptible to sunburn) might derive greater relative benefit from augmenting their intrinsic antioxidant defenses through dietary sources. Future clinical studies must therefore stratify participants or analyze outcomes by these variables to determine if personalized dietary or nutraceutical approaches for skin health merit consideration.

Beyond inter-individual variation, the translational potential of these promising dietary phytochemicals, particularly for oral administration in functional foods, is fundamentally constrained by intrinsic pharmacokinetic challenges. Key compounds like curcumin and resveratrol are widely recognized for their low systemic bioavailability due to extensive first-pass metabolism, rapid conjugation (glucuronidation and sulfation), and swift clearance from circulation [101,102]. These processes drastically reduce the proportion of the active compound that reaches peripheral tissues like the skin. Consequently, the potent anti-senescence effects observed in vitro and in some direct-application in vivo models may not directly translate to equivalent efficacy through dietary intake. Addressing these limitations—through strategies such as bioavailability enhancers (e.g., piperine with curcumin), structural analogs, advanced delivery systems (e.g., nano-formulations, phytosomes), or topical delivery systems—is therefore a critical frontier for realizing their practical application in skin-health-promoting nutraceuticals.

To address the aforementioned bioavailability challenges, various solutions have been proposed in food science and pharmaceutics. To overcome these bioavailability barriers, advanced formulation and delivery strategies have been actively explored. Nano-formulations, such as liposomes, nanoemulsions, and polymeric nanoparticles, can protect bioactive compounds from degradation, enhance intestinal absorption, and facilitate targeted delivery. For instance, curcumin-loaded nanoliposomes have demonstrated significantly improved oral bioavailability, as evidenced by enhanced bioaccessibility in simulated digestion studies [101]. Novel particle engineering approaches also markedly increase intestinal cell transportation [103]. Fermentation processes, particularly by gut microbiota, biotransform curcumin into metabolites with greater bioavailability [104]. Similarly, phospholipid complexes (phytosomes)—as critically reviewed for silymarin and curcumin—are established technology to markedly improve bioavailability and cellular uptake compared to standard extracts [105]. These delivery systems represent promising avenues to bridge the gap between the potent bioactivity observed in vitro and the efficacy required for systemic, dietary approaches to skin health.

Specifically for functional food development, these delivery strategies can be further integrated with food matrices. Translating the promising NRF2-activating dietary phytochemicals discussed above (e.g., curcumin, resveratrol) into effective functional food ingredients necessitates strategies to overcome their inherent instability and low bioavailability. Fortunately, contemporary food science offers several viable encapsulation and delivery solutions. Nano-encapsulation using food-grade biopolymers, such as zein/carboxymethyl chitosan nanoparticles, has been proven to co-deliver curcumin and resveratrol efficiently, significantly improving their aqueous stability, antioxidant activity retention, and bioaccessibility during simulated digestion. This approach aligns with the broader consensus that nanotechnology can enhance the stability and bioavailability of herbal bioactives [106]. Furthermore, advanced food-grade delivery systems like emulsion-filled hydrogels or water-in-oil-in-water (W1/O/W2) double emulsion gels can encapsulate hydrophobic compounds, providing robust protection against environmental stressors and enabling controlled, sustained release in the gastrointestinal tract [107,108]. Lastly, leveraging intrinsic molecular interactions, such as the formation of stable complexes between polyphenols and peptides, can shield bioactives from enzymatic degradation and preserve their activity throughout digestion [109]. These technological pathways provide a realistic foundation for developing functional foods that retain the stability and potency of phytochemicals targeting skin health via the NRF2 pathway.

Building upon the above analysis, we propose a clear translational pathway to guide future research. The compelling pre-clinical evidence summarized herein calls for a translational pathway to evaluate NRF2-targeting phytochemicals in human skin health. The most immediate and promising next steps could follow a sequential approach: Conducting human pharmacokinetic and proof-of-concept safety/efficacy trials using bioavailability-enhanced formulations (e.g., nano-curcumin, phytosomes) is a critical first step. Subsequently, early-phase efficacy trials may integrate mechanistic biomarkers (e.g., NRF2 target gene expression in skin) alongside clinical assessments of photoaging to validate the target engagement in humans. Furthermore, long-term studies and investigations into synergistic combinations of these dietary compounds could unlock potent, low-dose interventions suitable for integration into functional foods or nutraceuticals for sustainable skin health promotion.

## 6. Conclusions

This review elucidates the central role of the NRF2 signaling pathway in counteracting skin aging, demonstrating that its age-related decline underlies increased oxidative stress, chronic inflammation, and mitochondrial dysfunction. Importantly, emerging clinical evidence supports the translational relevance of modulating this pathway and informs key considerations for its dietary targeting. Integrated analysis of literature and transcriptomic datasets reveals that dietary phytochemicals such as curcumin, resveratrol, and sulforaphane can activate NRF2, often via competitive binding with KEAP1, thereby enhancing cellular antioxidant defenses and mitigating key drivers of senescence. These findings provide a mechanistic foundation for developing NRF2-targeting nutraceuticals and functional foods, although translational challenges including bioavailability and skin-type variability must be addressed to realize their full potential in dietary strategies for skin health.

## Data Availability

No new data were created or analyzed in this study.

## Supplementary Materials

The following supporting information can be downloaded at https://www.mdpi.com/article/10.3390/biology15010039/s1, Figure S1: Comparative analysis of STRING and GO-BP enrichment results; Table S1: Differential expression of NFE2L2 target genes involved in iron homeostasis, thioredoxin function, and antioxidant pathways following NRF2 knockdown; Table S2. Component-Kelch molecular docking.

## Abbreviations

The following abbreviations are used in this manuscript:

ARE, antioxidant response element; caNrf2, constitutively active Nrf2 mutant; CAT, catalase; CO, carbon monoxide; DDR, DNA damage response; DEGs, differentially expressed genes; DFs, dermal fibroblasts; ERK, extracellular signal-regulated kinase; GPx, glutathione peroxidase; HMOX1, heme oxygenase 1; H_2_O_2_, hydrogen peroxide; HO-1, heme oxygenase-1; IL-1β, interleukin-1 beta; JKN, c-Jun N-terminal kinase; KEAP1, Kelch-like ECH-associated protein 1; MAPS, mitogen-activated protein kinase; MDA, malondialdehyde; mtDNA, mitochondrial DNA; NQO1, NAD(P)H quinone dehydrogenase 1; NRF2, nuclear factor erythroid 2-related factor 2; O2•^−^, superoxide anion; OH•, hydroxyl radical; PGC-1α, peroxisome proliferator-activated receptor gamma coactivator 1-alpha; PLR, Puerariae lobatae radix; ROS, reactive oxygen species; Sal-B, Salvianolic acid B; SASP, senescence-associated secretory phenotype; SFN, Sulforaphane; sMAF, small musculoaponeurotic fibrosarcoma; SOD1, superoxide dismutase 1; TCM, Traditional Chinese Medicine; TNF-α, tumor necrosis factor-alpha; UV, ultraviolet; UVA, ultraviolet A; UVB, ultraviolet B.
