# Supplementary material for: Dietary Phytochemicals Targeting NRF2 Against Skin Cellular Senescence: Mechanistic Insights and Potential for Functional Food Development
Source: Biology (Basel). 2025 Dec 25;15(1):39. doi: 10.3390/biology15010039 (PMC12785134; doi:10.3390/biology15010039)
Supplement: Supplementary file 1 [file biology-15-00039-s001.zip › biology-4035799-supplementary.pdf]

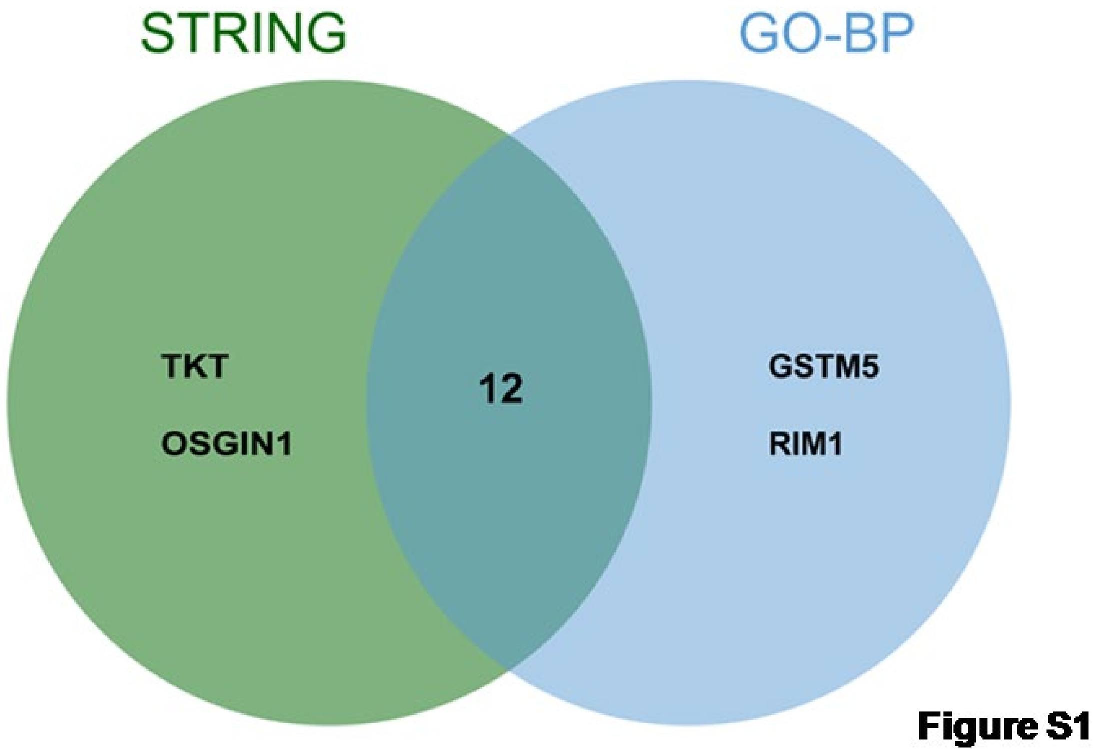

Figure S1: Comparative analysis of STRING and GO-BP enrichment results.

Table S1. Differential expression of NFE2L2 target genes involved in iron homeostasis, thioredoxin function, and antioxidant pathways following NRF2 knockdown.

	Maintaining iron homeostasis				Maintaining the function of thioredoxin		Other antioxidant pathways						
**log2FC**	FTH1	FTL	HMOX1	SLC7A11	SRXN1	TXNRD1	G6PD	MT1E	NQO1	OSGIN1	SLC7A11	SOD3	TKT
**si-1**	-1.15*	-2.13***	-1.75**	-1.12**	-1.72***	-2.52***	-1.44**	-1.27*	-3.56***	-2.84***	-1.12**	-1.40**	-1.82***
**si-2**	-1.55***	-1.83***	-1.72**	-1.32**	-1.06***	-2.71***	-1.28***	-1.30**	-3.61***	-1.92***	-1.32**	-1.23**	-1.48***

*, $P < 0.05$; **, $P < 0.01$; ***, $P < 0.001$; compared to the control group.

Table S2. Component-Kelch molecular docking.

Component	Binding energy/(kcal/mol)	Binding site
Curcumin	-4.02±0.16	ASN-469
Genistein	-6.61±0.36	VAL-418<br>LEU-365<br>ILE-559
Resveratrol	-6.90±0.35	VAL-418<br>VAL-514<br>VAL-604
Sulforaphane	-4.83±0.02	VAL-465<br>VAL-512<br>VAL-606<br>GLY-367
Zerumbone	-7.14±0.05	VAL-467
